# Advancing cross-disciplinarity in bone and joint infection science using the COMBINE approach: an example from Denmark

**DOI:** 10.5194/jbji-10-1-2025

**Published:** 2025-01-09

**Authors:** Louise Kruse Jensen, Thomas Bjarnsholt, Hans Gottlieb, Mats Bue

**Affiliations:** 1 Department of Veterinary and Animal Sciences, University of Copenhagen, Frederiksberg, Denmark; 2 Costerton Biofilm Center, Department of Immunology and Microbiology, University of Copenhagen, Copenhagen, Denmark; 3 Department of Clinical Microbiology, Copenhagen University Hospital, Rigshospitalet, Copenhagen, Denmark; 4 Department of Orthopaedic Surgery, Herlev Hospital, Herlev, Denmark; 5 Department of Clinical Medicine, Faculty of Health, Aarhus University, Aarhus, Denmark; 6 Department of Orthopaedic Surgery, Aarhus University Hospital, Aarhus, Denmark

## Abstract

In 2018, the Centrum fOr translational Medicine on Bone and joint INfEctions (COMBINE) was created to facilitate collaboration among Danish scientists and researchers dedicated to bone and joint infection research. The COMBINE approach was developed to ensure successful collaboration, and this publication aims to share this successful approach.

## 1 Introduction

In the coming years, there is expected to be a significant increase in patients suffering from bone and joint infection (BJI) (Metsemakers et al., 2024; Patel, 2023). This increase is expected across all types of BJIs, including diabetic foot osteomyelitis, fracture-related infection (FRI), prosthetic joint infection (PJI), and chronic osteomyelitis. The rise is driven by a growing number of primary surgeries, an increasing population of elderly patients, and more co-morbidities such as diabetes or immunosuppression (Konstantinos, 2017). BJIs impose similar individual and socioeconomic burdens to cancer, including pain, reduced mobility, prolonged hospitalization, diminished quality of life, and substantial healthcare costs (Metsemakers et al., 2024; Patel, 2023; Walter et al., 2021). For example, the 5-year survival rate of patients with PJI is lower than that of individuals diagnosed with breast cancer, prostate cancer, or malignant melanoma (Zmistowski et al., 2013; Sandiford et al., 2021). However, cancer treatment has seen remarkable progress over the past few decades and has rightfully received significant attention, whereas BJI has remained a silent disease (i.e. no famous social media ambassadors, no public awareness, no political or medical agenda focus, and limited funding possibilities). This is despite BJI patients reporting significantly impaired quality of life, often even worse than patients with a broad variety of cancers (Hotchen et al., 2023; Walter et al., 2021). Furthermore, the menace of antimicrobial resistance is steadily rising and has been identified by the World Health Organization (WHO) as one of the top five global public health challenges (Antimicrobial Resistance Collaborators, 2022). Thus, we are witnessing a delicate situation, the severity of which is massive: BJI incidence is drastically increasing, and killing the bacterial agents associated with these infections may be extremely challenging in the future.

Solutions to the challenges posed by BJIs depend on collaboration between disciplines within and beyond medical science. Within medical science, BJI diagnosis is based on a combination of clinical signs or symptoms, laboratory findings, and imaging. Treatment usually requires major surgical procedures and prolonged antimicrobial treatment. Therefore, collaboration between orthopaedic surgeons, plastic surgeons, infectious diseases specialists, clinical microbiologists, radiologists, and pathologists constitutes the clinical cornerstone of the optimization of BJI management (Ferguson et al., 2021). Beyond medical science, a fundamental understanding of disease mechanisms; novel drug, device, biocomposite, and 3D-printing development; and solutions to antimicrobial resistance are sought through a combination of laboratory work and in vivo models. Basic scientists with a non-medical background (e.g. bioengineers, molecular biologists, veterinarians, chemists, microbiologists, and pharmacologists) are often responsible for these solutions. In this context, it is important to remember that breakthroughs inevitably come from unexpected places and odd juxtapositions based upon a deep understanding of the specific core biology in its broadest sense (Sfeir et al., 2022). Clearly, the complexity of BJIs challenges the traditional disciplinary silo training and thinking. It is no longer enough to just be an orthopaedic surgeon or a microbiologist when problems involve almost unlimited complexity. Therefore, there is a critical need for more engaged cross-disciplinarity in BJI research. A cross-disciplinary approach enables an interchange of knowledge and experience, which can stimulate innovative solutions to challenges. Moreover, such an approach plays a pivotal role in disseminating knowledge into practice and policy (Ding et al., 2020). Despite good intentions, cross-disciplinarity is not as easy as it sounds and can be difficult to operationalize (Ding et al., 2020; Aagaard-Hansen, 2007). Collaboration issues can be hindered by a lack of knowledge, conflicting standards, different methodologies, or simply negative attitudes and prejudices (Aagaard-Hansen, 2007).

To foster cross-disciplinarity in BJI research, the authors created the Centrum fOr translational Medicine on Bone and joint INfEctions (COMBINE) in 2018. The idea of the COMBINE initiative was to bring all kinds of Danish scientists and researchers working with BJI research together and to increase collaboration between medical specialties. The COMBINE approach for successful togetherness within and between basic and clinical disciplines has been developed through meetings involving knowledge sharing, panel debates, and networking. Via this publication, we aim to pass on the COMBINE approach, share our experiences, and inspire basic scientists and clinicians to work more closely together. Therefore, the COMBINE aim, working methods, values, and outcomes are reported in the following.

BJIs have historically been the domain of orthopaedic surgeons. Nonetheless, the groundbreaking discoveries of bacteria in osteomyelitis by Louis Pasteur (1860), antiseptics by Joseph Lister (1867), and penicillin by Alexander Fleming (1929) more than a century ago underscored the essential interplay between BJI and the domains of clinical microbiology and infectious diseases (Abulfotooh, 2003). The study of disease biology has traditionally been compartmentalized into distinct silos defined by educational and medical specialties (Kuhn, 2012) – a construct deeply embedded in the human desire to systematize and structure nature. Despite the obvious inherently cross-disciplinary nature of BJI, it was not until recently that the clinical value of multidisciplinary team (MDT) approaches in BJI was fully recognized (Bauer et al., 2012; Broom et al., 2023; Carlson et al., 2020; Dudareva et al., 2017; Ferguson et al., 2021; Ntalos et al., 2019; Rupp et al., 2023). Today, many specialized BJI units have emerged worldwide, and the European Bone and Joint Infection Society (EBJIS) has formally recognized the vital importance of collaborative efforts by extending its membership beyond surgeons to all clinicians and scientists working in the field of BJI (Walenkamp, 2018). Several BJI institutions have also expanded further to collaborate more intensively with basic scientists, and sessions focused on basic science have gained significant interest at annual EBJIS meetings. Consequently, the COMBINE approach is not unique: various methods have been developed to bridge basic and clinical science. The COMBINE strategy builds upon the inspirational work of pioneering individuals and institutions and offers a framework for initiating or enhancing cross-disciplinarity in BJI. Nevertheless, achieving true cross-disciplinarity is more challenging than it may seem, and the COMBINE approach serves as a valuable tool to emphasize this and advocate for success.

**Figure 1:**
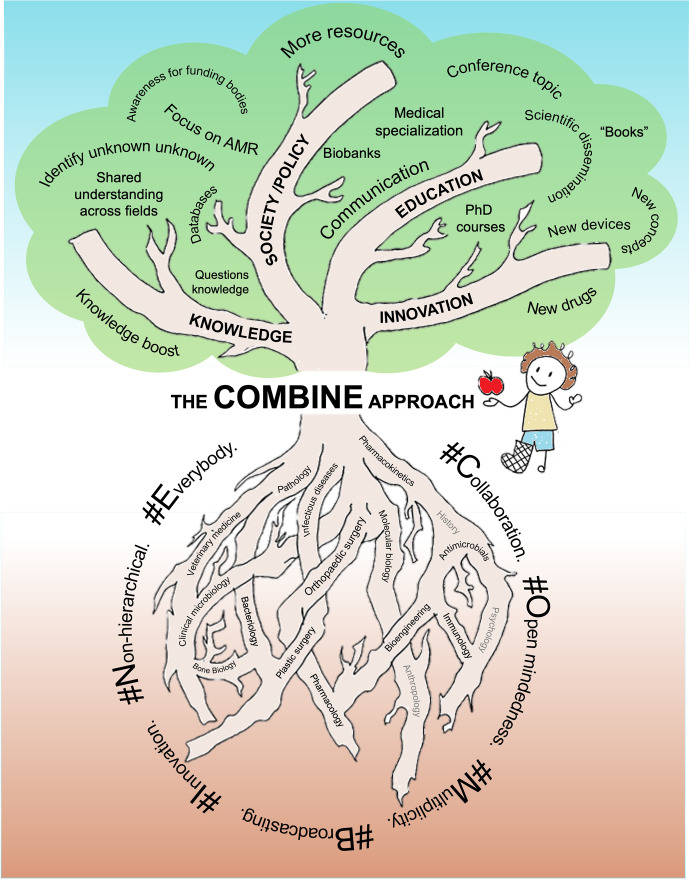
**Figure 1**The Centrum fOr translational Medicine on Bone and joint INfEctions (COMBINE) aims to bring all kinds of scientists and researchers working with bone and joint infections together and to increase collaboration between medical specialties. So far, the disciplines depicted using black text in the roots of the diagram have been enrolled, whereas those depicted using grey text represent unexplored possibilities. The “#Values” are the core of COMBINE. The COMBINE approach impacts knowledge, innovation, and education and also shapes social/political aspects. The ultimate fruitful success (the apple) of COMBINE is improved clinical outcomes for BJI patients.

## 2 COMBINE aim

The overall aim of COMBINE is to add another dimension to the existing pure basic and clinical science silo thinking by sharing knowledge and networking (Fig. 1). By sharing factual knowledge from clinicians explaining clinical cases, situations, and problems or from basic scientists explaining bone biology, antimicrobial chemistry, bacterial metabolism, etc., it is possible to achieve mutual understandings. Furthermore, sharing knowledge about ongoing activities, funding plans, methodology, in-house technical equipment, theories, paradigms, and historical aspects of all involved disciplines is essential to promote growth and breakthroughs. Knowledge sharing facilitates new thinking, serendipity, and networking, bridging ongoing and future collaboration. COMBINE activity in clinical MDT units aims to produce common treatment strategies, challenging the dogmatic and sometimes non-evidence-based way of approaching BJI and moving towards settings that can measure outcomes, compare data, and learn from each other.

## 3 COMBINE methods

The prime COMBINE method is an open-minded, non-hierarchal togetherness. The foundation for togetherness is achieved by connecting interested participants from hospital and university networks from all parts of Denmark. The concept for the meetings includes the invitation of international speakers, presentations of research projects (including obstacles and current problems), new ideas for paradigm shifts, the discussion of high-impact papers, or panel debates on selected topics, all followed by plenty of time to mingle and talk. Presentations are equally shared between basic and clinical scientists at different career levels (from master's students to professors). All presentations are followed by obligate feedback and discussion for inspiration. Especially when this comes from another scientific area, it can provide perspective on the standard conceptual thinking in a field. There is no apparent structure or hierarchy in COMBINE, nor is there an overall research project or ownership; it is intended that all can initiate collaboration without any obligation to the COMBINE board. To date, the meetings have succeeded in bringing the following disciplines together: orthopaedic surgeons, plastic surgeons, clinical microbiologists, infectious disease specialists, physicians, veterinarians, bioengineers, molecular biologists, immunologists, biologists, chemists, bone biologists, and microbiologists (Fig. 1).

## 4 COMBINE values

The most important part of COMBINE is the “#Values”, which set the atmosphere at the meetings and aid in collaboration (Fig. 1). These values are the core of COMBINE and are undoubtedly the reason for its success. The aforementioned #Values are as follows:

# *Collaboration and cross-disciplinarity*. Support and promote the creative and unbiased application of knowledge and skills from one field or discipline to others.
# *Open-mindedness and shared curiosity*. Promote an interest in dealing with both the known unknown and unknown unknown.
# *Multiplicity*. As diversity brings insight, take the time to understand other disciplines. All disciplines have individual organizational cultures, settings, and rules. Understanding these is crucial to respect the position of collaborators from other fields.
# *Broadcasting*. Promote the curious dissemination of ideas and knowledge within and beyond COMBINE. Encourage a willingness to meet and present outside of scientific comfort zones.
# *Innovation*. Establish ideas, no matter how farfetched, incomprehensive, or controversial they may initially appear. Broadening the perspective of problem-solving is needed to overcome difficult clinical or scientific obstacles and induce serendipity.
# *Non-hierarchical structure*. Promote a trustworthy community and minimize academic and discipline hierarchies and rivalry. COMBINE holds no rights of possession for ideas or projects. All ideas can be shared without fear of being negatively exposed or overtaken.
# *Everybody can contribute*. Motivate participants to explore new ideas and be willing to learn from other disciplines. Participants should be humble when they work outside their own disciplinary boundaries.

## 5 Specific COMBINE outcome

Since its inception, the COMBINE approach has resulted in several scientific papers, conference presentations, and research grants on diverse aspects of BJI research. The authors of these different types of dissemination have been a mix of medical doctors and basic scientists (with at least three different educational backgrounds). Recently, joint PhD projects and international collaboration have also been established. Within the clinical field in Denmark, COMBINE has emphasized the need for in-hospital MDT teams to diagnose and treat BJI effectively. Strict MDT protocols and algorithms were formally commenced in 2018 at a designated BJI centre in Copenhagen. Recognizing that the process of implementing and organizing changes within organizations requires a significant temporal investment, strategies are now gradually being disseminated to additional institutions. Overall, the COMBINE approach has raised awareness of BJI in Denmark, leading to several invitations to organize COMBINE sessions and symposiums at national and international meetings and conferences, also with no obvious connection to the BJI field. In general, BJI patient care, patient outcomes, and science has moved forward in Denmark due to the COMBINE approach.

## 6 Conclusion and perspective

We have demonstrated that the COMBINE approach can enhance networking, knowledge sharing, dissemination, and the overall output of BJI research. Initiatives like COMBINE are pivotal for future bottom-up growth in the field of BJI research. From a broader perspective, the COMBINE approach contributes to advancing knowledge, fostering innovation, enriching education, and shaping political/societal aspects (Fig. 1). The ultimate fruitful success of COMBINE is improved clinical outcomes for BJI patients. The time gap between the apple and improved patient outcomes can vary significantly depending on the basic and/or clinical science levels involved (Fig. 1). In the future, we plan to strengthen COMBINE and further broaden BJI horizons by inviting humanity and social science disciplines, such as anthropology, sociology, psychology, and history, so that we can gain a deeper insight into the ancient, present, and future BJI patient.

In the forthcoming years, it is fundamental to undertake an objective evaluation of the outcomes associated with basic clinical cross-disciplinary initiatives, although all of the positive effects seem logical. Objective parameters of interest include the quantity of research publications, patents, granted funds, and clinical guidelines (and their respective efficacy). Such evaluations will be relevant not only for the COMBINE approach but also to established institutions with solid cross-disciplinary research traditions as well as new research groups, regardless of whether they derive inspiration from COMBINE or from other frameworks. A primary advantage of quantifiable outputs lies in their potential to justify increased funding opportunities and draw political attention to the field of BJI. A viable methodology for measuring future outputs involves the establishment of national or regional registries dedicated to basic clinical cross-disciplinary projects. Such strategic approaches could guarantee long-term follow-up and facilitate precise impact assessments, ensuring adequate and effective progress tracking.

We hope that this publication will be used as a lens for understanding and discussing successful approaches that foster cross-disciplinarity in BJI science.

## Data Availability

No data sets were used in this article.
